# Vaping-associated Lung Injury

**DOI:** 10.7759/cureus.6216

**Published:** 2019-11-22

**Authors:** Ghulam Aftab, Mudassar Ahmad, Douglas Frenia

**Affiliations:** 1 Internal Medicine, Orange Park Medical Center, Orange Park, USA; 2 Pulmonary, Saint Peter’s University Hospital, New Brunswick, USA; 3 Pulmonary Critical Care, Saint Peter’s University Hospital, New Brunswick, USA

**Keywords:** vaping, lung injury

## Abstract

Many cases related to vaping-associated lung injury have recently been reported to the Center for Disease Control (CDC). It is, therefore, important for clinicians to be aware of this disease.

Here, we present the case of a 46-year-old female patient, who had recently started vaping. She presented to the hospital with dyspnea; since her condition deteriorated quickly, she was mechanically ventilated for acute respiratory failure. When a computed tomography angiography (CTA) chest was performed, patchy alveolar opacities were seen throughout both lungs. The patient’s workup for infectious and cardiac etiology was negative. She was diagnosed with vaping-associated lung injury. Later, she recovered and was discharged to a rehabilitation center.

## Introduction

In recent times, adult smokers have been transitioning to the use of e-cigarettes. At present, there are approximately 4 million Americans using e-cigarettes, or vaping products [1]. As of October 8th 2019, the Center for Disease Control (CDC) had reported 1080 cases of vaping-associated lung injury from across America - since then, this number continues to grow [2]. In addition, 26 deaths likely linked with the same disease have also been reported. Even though the CDC and FDA are not aware of the precise etiology of lung injuries reported in these cases, the only common thread across all is that all patients used either e-cigarettes or vaping products. In light of this information, it is important that patients and clinicians be prepared with the tools required to diagnose and manage this disease.

## Case presentation

A 46-year-old female with a history of asthma comes with a chief complaint of worsening shortness of breath presented with associated dry cough for two days. She reports minimal exertion made her dyspnea worse. She denies recent travel, sick contact, fever, chills, night sweat, chest pain and sputum production, as well as prior history of lung disease. She states that she has never smoked or used vaping products. She reports a remote history of using marijuana and cocaine.

Upon physical examination, the patient had hypoxia on room air. She was tachypneic and using respiratory accessory muscles, though was able to speak in full sentences.

A computed tomography angiography (CTA) chest was performed which showed diffuse patchy alveolar opacities throughout both lungs. The patient was initially placed on high flow nasal cannula and broad spectrum antibiotics, but her condition worsened quickly - she had to be intubated and temporarily paralyzed to help with oxygenation. Her infectious workup serologies and bronchial alveolar lavage analysis was negative. She was started on high dose steroids due to concern for acute interstitial lung disease. Subsequent workup for rheumatologic and cardiac cause was negative.

Two days post intubation, the patient’s mother revealed to the nursing staff that the patient, contrary to what she admitted to the hospital staff earlier, had in fact been using e-cigarettes one month prior to her hospital admission. Meanwhile, the patient’s condition improved, until she was extubated to nasal cannula after being on the ventilator for five days. She was later transitioned to room air and discharged to a rehabilitation center. She was advised to complete a ten-day long course of steroids.

Investigations

Upon arrival, the patient had a complete blood count performed. She had an elevated white blood count with bandemia, as well as an elevated lactic acid of 2.3 mmol/L. Her CD4 count was low, but she tested negative for HIV. Blood cultures were drawn and were negative. Respiratory viral panel and influenza testing was negative. Urine legionella and streptococcus antigen were negative.

A basic rheumatologic workup was performed. She was found to have an antinuclear antibodies titer of 1:40. Her tests for rheumatoid factor and antinuclear cytoplasmic antibodies turned out negative.

**Figure 1:**
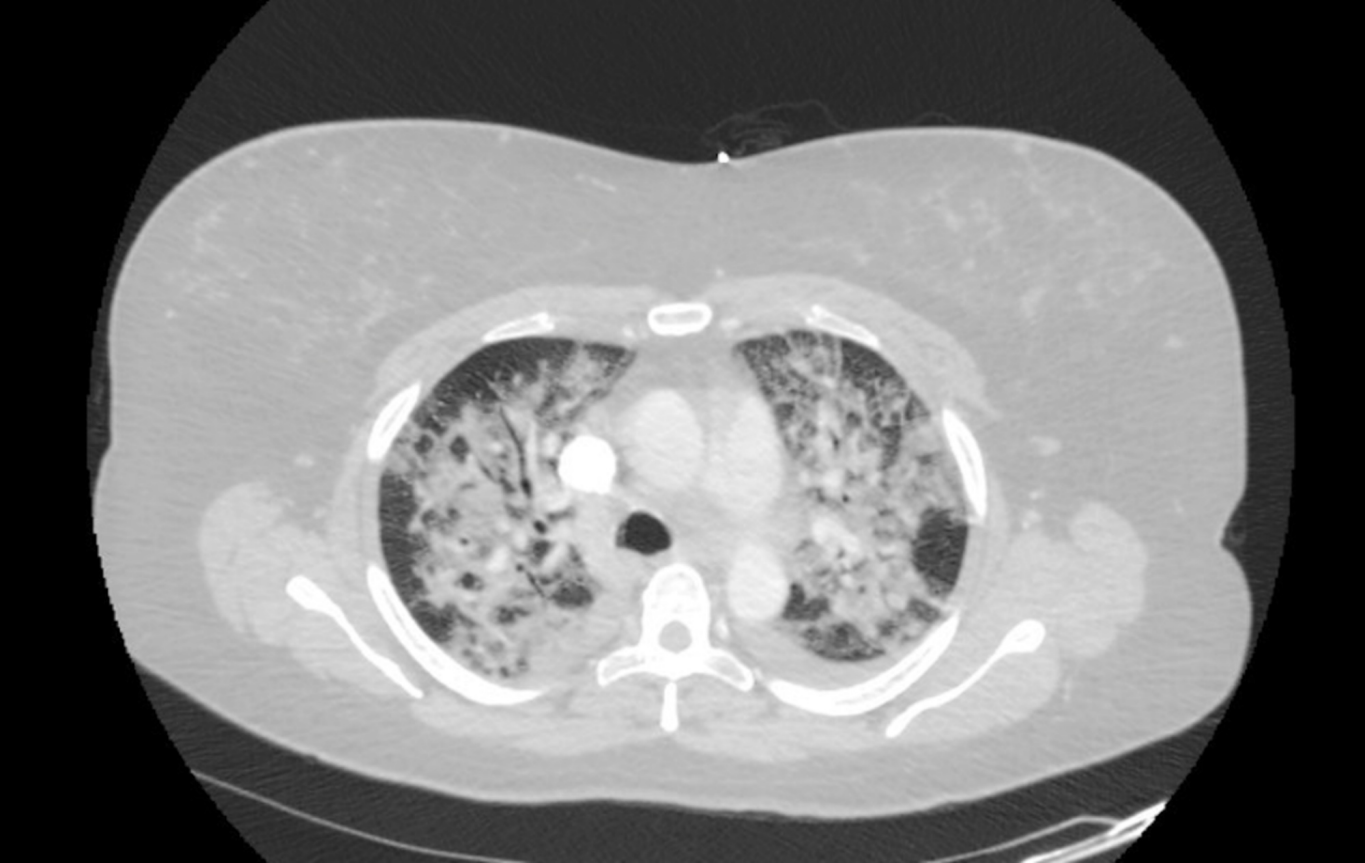
CTA chest showing bilateral alveolar opacities. CTA: Computed tomography angiography

Echocardiogram showed normal ejection fraction and there was no valvular abnormality. CTA chest did not show a pulmonary embolism, however it showed bilateral consolidation throughout both lungs (Figure 1).

**Figure 2:**
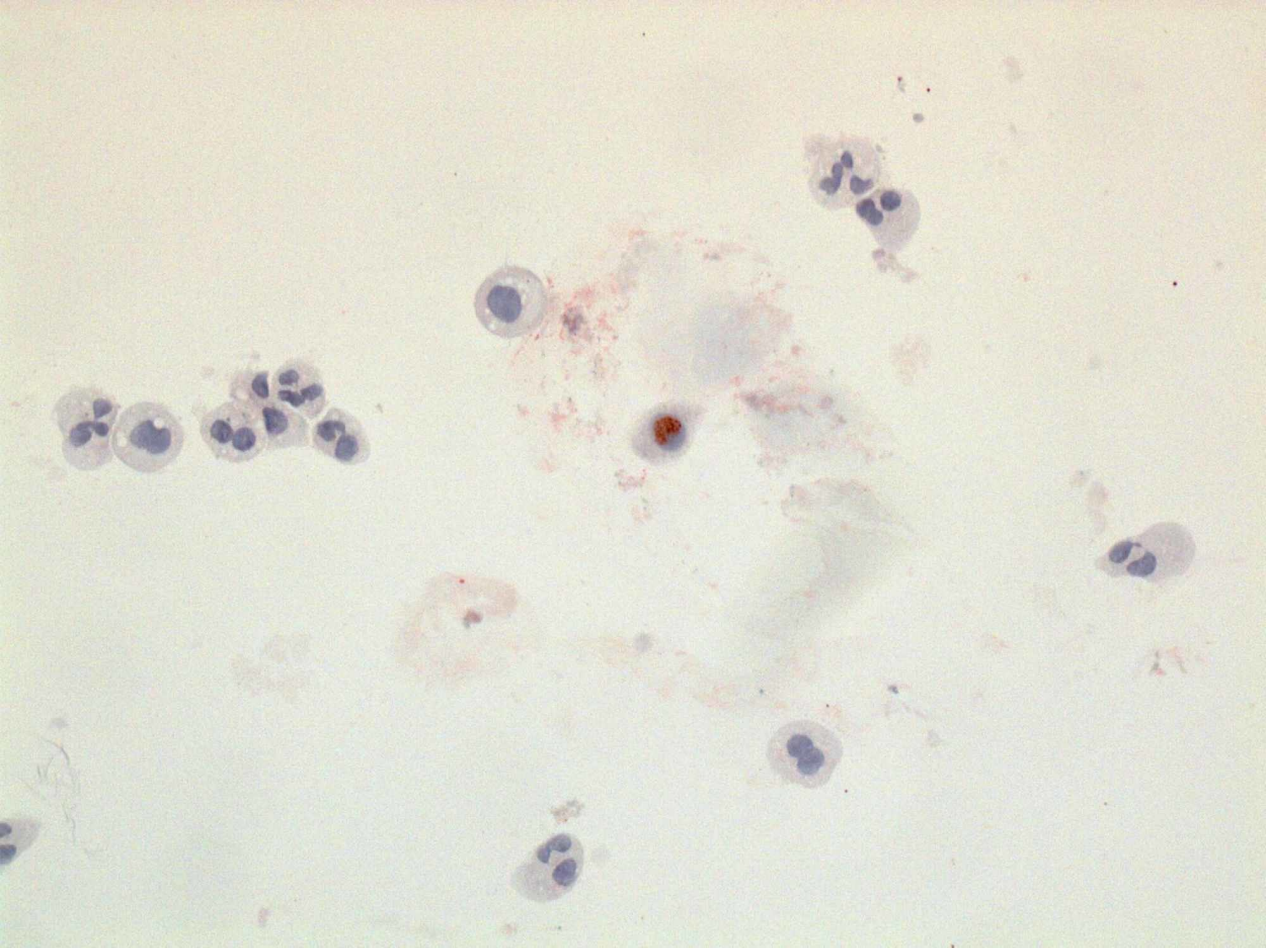
Oil Red O stained pulmonary macrophage (center of the picture), surrounded by pulmonary macrophages not stained by Oil Red O stain.

A fibreoptic bronchoscopy with bronchoalveolar lavage (BAL) was performed. BAL analysis showed the patient had 91% neutrophils. Cultures from the BAL fluid were negative. No cysts of pneumocystis were identified. Oil Red O stain was performed and it showed positive staining in a small number of alveolar macrophages (<5% of the cellular population present) (Figure 2).

Differential diagnosis

Initially, the patient was thought to have community acquired pneumonia as she had an elevated white blood cell count with bandemia, and her CT chest showed bilateral consolidation. Because of the patient’s low CD4 count, we suspected she may have pneumocystis jirovecii pneumonia. Upon BAL fluid testing, however, no cysts of pneumocystis were identified. Additionally, the patient tested negative for HIV and no infectious etiology was identified after an intensive workup; blood culture, viral panel and culture on the BAL fluid were also negative.

As the patient had had bilateral infiltrates, there was concern for heart failure. When an echocardiogram was performed, her ejection fraction was found to be 60%, and there were no valvular abnormalities.

Diffuse alveolar hemorrhage was also in the differential, but antineutrophil cytoplasmic antibodies (ANCA) testing was negative. Since the BAL did not return bloody fluid, this finding reduced the likelihood of diffuse alveolar hemorrhage.

Certain interstitial lung diseases may present in a similar manner. In eosinophilic pneumonia the BAL has greater than 25% eosinophils [3]. Our patient had 0% eosinophils.

When it was later discovered that the patient had started vaping one month prior to admission, the BAL fluid was tested for Oil Red O stain. Our patient had minimal Oil Red O stain macrophages, in line with other vaping-associated lung injury cases that were seen.

In light of the patient’s history and our systematic ruling out of infectious, rheumatologic and cardiac causes, the patient was deemed to have vaping-induced lung injury. The patient met the CDC case definition for a confirmed case.

Treatment

The patient was started on an antibiotic course of Ceftriaxone and Azithromycin, which was later broadened to Vancomycin and Piperacillin/Tazobactam when she was intubated. She was also started on high dose Sulfamethoxazole/Trimethoprim due to her low CD4 count. When she developed acute respiratory distress syndrome, with worsening hypoxemia, she was placed on high dose steroids. This was also in part due to concern for acute interstitial lung disease. She was placed on low tidal volume ventilation per ARDSnet protocol. Though no randomized clinical trial has been performed, a recent article shows that most patients with vaping-associated lung injury are treated with steroids, and have responded to them [4]. Antibiotics and Sulfamethoxazole/Trimethoprim were later stopped as infectious workup was negative.

Outcome and follow-up

The patient remained in the hospital for 12 days. Later, she was discharged to a rehabilitation center. At the time of discharge, the patient was able to participate in physical therapy and continued to improve. On follow-up in the clinic the patient continued to improve.

## Discussion

Our patient met the criteria for definite vaping-induced lung injury (VILI), which comprises the following [5]:

1.) Use of vaping or dabbing in the 90 days before symptom onset,

2.) Pulmonary infiltrate on the chest X-ray or ground glass opacities on the chest CT scan,

3.) Absence of pulmonary infection in the initial workup,

4.) No evidence of an alternative cause.

Most vaping-associated lung injury patients are known to have used nicotine products, and tetrahydrocannabinol (THC) or cannabidiol (CBD) products. CDC has especially recommended not to use THC-based vaping products, even though the exact etiology causing the injury process is still unknown. Because of the health hazards posed by such products, though, it is imperative to regulate their use.

All patients diagnosed with vaping-induced lung injury to date have had bilateral pulmonary infiltrates - our patient’s case was similar. Many patterns of lung injury have been reported; however, ground glass opacities with subpleural sparing has been the predominant pattern [5].

When vaping-associated lung injury patients had Oil Red O stain performed on the BAL fluid, foamy macrophages ­were considered an indication of positive staining. Our patient’s Oil Red O stain also showed foamy macrophages. However, only a limited number of patients have had this test performed to date; because of the small sample size, this test may only be used as a marker of exposure, and not of disease. A transbronchial biopsy may give more information about the pattern of lung injury. Corticosteroids have been used on most VILI patients, and they are known to improve patient condition.

## Conclusions

Vaping history is important especially in patients presenting with dyspnea. Vaping-associated lung injury should not be diagnosed without ruling out infectious causes, especially during the flu season. Clinicians should not recommend e-cigarettes as alternatives to traditional cigarette smoking. Bronchoscopy with BAL may be used to evaluate lipid laden macrophages, but its real utility is still being debated. Steroids have been shown to be beneficial in most cases.
